# Supplementary material for: The oldest Pottery Neolithic (PN) culture of northeastern Iran: First absolute dating from eastern Mazandaran plains
Source: PLoS One. 2025 Jun 10;20(6):e0322478. doi: 10.1371/journal.pone.0322478 (PMC12151361; doi:10.1371/journal.pone.0322478)
Supplement: Attachment — Submitted filename: Response to Reviewers.docx [file pone.0322478.s002.docx]

Answers to editors:

We wish to show you our gratefulness for addressing the issues in the paper. We tried our best to fulfil your requests and suggestions.

1. As recommended and after reading the Plos One template pdf, we correct the paper styles to the journal`s requirement.
2. As required by the editor we provide information of the organization who gave permission to us for field works.
3. We did not used any human, plant our animal or genetic samples in our paper, however, any other data that required by editor is available.
4. After carefully read the online page, we believe our paper does not need PLOS’ questionnaire on inclusivity in global research given that our research does not include the condition that required by this section at the journal.
5. As you required, we changed the acknowledgment as the way that the journal required. The statement “The author(s) received no specific funding for this work” is correct and we also will mention the current statement in the cover letter as well. The first author (also the corresponding author) received funds from the University of Mazandaran and his research grant for only the fieldwork; the funding does not cover the publication fee.
6. We acknowledge that we agreed to let access to the data by the journal if required. We would like to state that ‘all relevant data are within the paper’.
7. After checking the permission in Google earth website, we notice that the mentioned figure does not need permission from Google earth.

<https://about.google/brand-resource-center/products-and-services/geo-guidelines/#google-earth>

Also, the “figure 3” was actually a GIS map, not a satellite image, however, we change the map to another one to avoid any issue.

8. As you required, we did check the reference list again and we can assure you all the references are currently available to use.

Answers to reviewers:

We are very thankful for your time and effort to read our manuscript and trying to help us for to improve it. We also are very grateful for your positive comments and understanding the importance of our paper; therefore, we tried our best to fulfil your requests and suggestions.

Reviewer #1

Thank you so much for your time and effort we

1. Thank you for noticing this issue. We would like to mention that the terms “WMSeC” refers to “Western Mound of Sang-e Chakhmaq” and “EMSeC” refers to “Eestern Mound of Sang-e Chakhmaq”. We used these abbreviations to avoid the long name of the site. Both these abbreviations were mentioned in manuscript at the first appearance.
2. Thank you for carefully observation the “dot motif” in pottery section and you are right; these round bubbles like motif are too large to be considered as dot and we notice it as well. However, because in her thesis, Coolidge, named it dot motif we tried to be loyal to the original name. Although what we will do to satisfy your requier is to let readers know that the name Coolidge given to these motives are “dot motif” but we think it is more proper to call it “Polka motif”.
3. Thank you for reminding us about the sites name that we incorrectly put “Tell”. This is very important point and we will change it to the correct version as you recommended.
4. Thank you for mentioning the misspelled. We correct it.

Reviewer #2

Thank you so much for your time and effort. We understand your concern about the language of the paper; therefore, we asked scholars at British universities to revise the language of the paper.
